# Brain localization and morphological changes in NREM parasomnias. A systematic review study

**DOI:** 10.1007/s11325-025-03492-z

**Published:** 2025-10-15

**Authors:** Mengesha S. Biresaw, József Vitrai, Peter Halász, Vivian M. Correa, Anna Szűcs

**Affiliations:** 1 Institute of Behavioural Sciences, Semmelweis University, Budapest, Hungary; 2 Department of Obstetrics and Gynaecology, Széchenyi István University, Győr, Hungary; 3 Szentágothai Doctoral School, Semmelweis University, Budapest, Hungary

**Keywords:** NREM parasomnias, Localization, Morphology, Sleepwalking, Sleep eating

## Abstract

**Background:**

Individuals with NREM parasomnias exhibit abnormal slow-wave activity and fragmented sleep. Sleep-state dissociation is the prevailing concept of NREM parasomnia-episodes; typically emerging from N3/N2 stages of NREM sleep’s first cycle at the turning-point of deep sleep and arousal. While these relations provide a frame to understand these conditions, their mechanism and brain-topography remain unclear.

**Methods:**

We performed a systematic search of the literature (1/01/2015-20/06/2024) on brain-topographies and morphological changes based on neurophysiological and imaging studies in patients with NREM parasomnias.

**Results:**

It was shown that immediately preceding clinical episodes, the EEG spectral power of delta and theta frequency-bands increased in parallel with its reduction in the cingulate, motor, and premotor/supplementary motor cortices. Far from clinical episodes, in NREM and REM sleep as well as in wakefulness, a cortico-cortical sleep-state dissociation occurred, too. In addition, the partial arousals of episodes evolved from ‘deeper’ sleep with lower-amplitude slow waves, compared to episode-free arousals of the same people with NREM parasomnias. A single MR-morphology study revealed decreased grey-matter volume in the left dorsal posterior cingulate and mid-cingulate cortices in patients with mixed NREM parasomnias.

**Conclusion:**

Based on recent research, the state-dissociation evidenced in clinical episodes might characterize each vigilance state of people with NREM parasomnias, even outside the episodes, making sleep-wake dissociation a trait-like core feature of NREM parasomnias. The anterior cingulo-frontal regions seem to have central roles.

PROSPERO registration ID: CRD42024552562.

**Supplementary Information:**

The online version contains supplementary material available at 10.1007/s11325-025-03492-z.

## Introduction

Parasomnias are a group of sleep disorders characterized by abnormal behavioural, experiential, or physiological events occurring during sleep, sleep-wake transitions, or arousals from sleep. These events may include complex movements, emotions, dreams, or autonomic nervous system activities and typically occur without full consciousness or memory to the episode [1, 2].

There are two main types based on the sleep stage during which they occur, even though there is a third category called other parasomnias that are not specific to sleep stage or are secondary to other conditions [3].

Non-rapid eye movement (NREM) sleep-related parasomnias, also termed Disorders of Arousal (DOA), occur during NREM sleep, typically in the first third of the night, when partial arousals arise from deep slow wave sleep. DOA are classified as sleepwalking (somnambulism), sleep terrors (night terrors), confusional arousals, sleep-related eating disorder (SRED), and sexsomnia. There is little data on the prevalence of SRED and sexsomnia. They are characterized by involuntary, fully or partially amnestic behaviours during incomplete arousals from sleep involving eating/drinking and sexual behaviours respectively [3–10]. Rapid eye movement (REM) sleep-related parasomnias include REM Sleep Behaviour Disorder (RBD), nightmare disorder, and recurrent isolated sleep paralysis, typically occurring in the second half of the night where REM sleep prevails. RBD manifests dream enactment behaviours due to the loss of normal REM sleep muscle atony [11]. Sleep paralysis is the opposite: muscle atony (paralysis) of REM sleep enduring or intruding between wakefulness and superficial sleep; often associated with fear or hallucinations [12].

DOA predominantly begins in childhood, with a prevalence ranging from 13 to 39%, and there is a significant prevalence decline with age [13–15]. It affects up to 34% of toddlers, 13.4% of school-age children [16], and 4.8% of adults [17]. In children, sleepwalking has a prevalence of 17%; night terrors have a prevalence of 39.8%, both decreasing to 2–4% in adulthood [18]. The lifetime and current prevalence of sleepwalking were 22.4% and 1.7%; of confusional arousals, 18.5% and 6.9%; and of SRED 4.5% and 2.2%, respectively [7, 19].

DOAs are often triggered by sleep-disturbing events or circumstances and emerge from the N2 or N3 stage of NREM sleep during the first sleep cycle at the turning point of deep sleep to arousal. An essential feature is sleep-wake dissociation during the episodes: certain brain parts are fully or partially awake, while the rest of the brain remains in slow-wave sleep [9, 20, 21]. Polysomnographic investigations have revealed that individuals with NREM parasomnias exhibited abnormal slow-wave activity and fragmented sleep [20, 22]. There were more arousals, including full awakenings following sleepwalking episodes, and a rise in slow wave sleep-fragmentation even far from them [23, 24]. In sleepwalking persons on episode-free nights, cyclic alternating pattern (CAP) A1 was reduced, involving decreased delta wave power during the first sleep cycles [25, 26].

Imaging studies, including SPECT, stereo-electroencephalography (EEG), and current source mapping in patients with DOAs, indicated a significant sleep state dissociation associated with the episodes. Deep slow-wave sleep in the fronto-dorsal regions and the whole cortex paralleled an activated, wake-like state in the anterior cingulate, anterior upper insular, and fronto-medial areas [27, 28]. An early influential study [29] on SPECT performed during sleepwalking found the activated region in the anterior cingulate region paralleling inactive (sleeping) regions elsewhere in the brain. Although DOA episodes have been linked to various brain regions (29), the involvement of the anterior cingulate and frontal areas seems typical based on most studies [21, 30].

An underlying sleep-dissociation is also supported by a systematic review indicating elevated beta in the anterior cingulate cortex paralleling enhanced slow-wave EEG activity just before the motor activations in frontal and central regions [20]. Similarly, a source localization analysis has revealed the increase of the beta frequency band in the anterior cingulate cortex during the last 4 s preceding behavioural episodes of DOA, suggesting the pre-episode arousal of those cortical areas involved in motor control [31].

Frontal and central hypersynchronous delta (HSD) (several continuous high-voltage delta waves ≥ 150 microV) in NREM sleep EEG has classically been considered a marker of DOA [32] while others deemed it a non-specific companion of arousals from NREM sleep [33]. HSD has occurred consistently after (33), before, or randomly relative to the episodes [34, 35].

In this systematic review, we focused on brain localization and EEG changes related to DOA and subtypes; rather than on clinical features. We aimed to answer the following questions.

1. Which brain areas or networks are involved in NREM parasomnias?
  - Are there specific brain areas or networks participating in the different subtypes?
2. Are there any brain-morphological changes underlying NREM parasomnias?
3. Is there any specific EEG change characterizing NREM parasomnias?

## Methods and materials

### Identification and selection of studies

We collected those studies that examined topographic and morphological changes in NREM parasomnias using EEG, MRI, fMRI, SPECT, and other neuroimaging techniques published between 01/01/2015 and 30/06/2024. Between 01 and 30/06/2024, we searched the following databases: PubMed/Medline, Science Direct, Google Scholar, and PsycINFO. An appropriate guideline for a systematic review and meta-analysis report was used, i.e., the Preferred Reporting Items for Systematic Reviews and Meta-Analyses (PRISMA-p) [36]. The primary search terms used in databases are shown below (Table 1).

**Table 1:** Primary search items used for databases

Database	Applied search items	Filter used
Google scholar	(Localization OR brain parts OR brain network OR Morphology changes OR EEG changes OR fMRI changes) AND (NREM parasomnias OR sleep-wake dissociation OR sleep walking OR sleep eating OR sleep talking OR sleep sex OR sexsomnia)	Study year 2015–2024
PubMed	((((((“brain mapping“[MeSH Terms]) OR (“brain waves“[MeSH Terms])) OR (“neuronal plasticity“[MeSH Terms])) OR (“anatomy category/abnormalities“[MeSH Terms])) OR (“electroencephalography“[MeSH Terms])) OR (“magnetic resonance imaging“[MeSH Terms]) AND ((y_10[Filter]) AND (fha[Filter]) AND (fft[Filter]))) AND (((((“parasomnias“[MeSH Terms]) OR (“sleep/analysis“[MeSH Terms])) OR (“somnambulism“[MeSH Terms])) OR (“dyssomnias“[MeSH Terms])) OR (“sleep wake disorders“[MeSH Terms]) AND ((y_10[Filter]) AND (fha[Filter]) AND (fft[Filter])))	Study year 2015–2024
PsycINFO	(Localization OR brain network OR Morphology changes AND NREM parasomnias OR sleep-wake dissociation OR sleep walking OR sleep eating OR sleep talking OR sleep sex OR sexsomnia)	Study year 2015–2024
Science Direct	(Localization OR brain parts OR brain network OR Morphology changes OR EEG changes OR fMRI changes) AND (NREM parasomnias OR sleep-wake dissociation OR sleep walking OR sleep eating OR sleep talking OR sleep sex OR sexsomnia)	Study year 2015–2024

### Eligibility criteria

All relevant English language research reports on brain topography or morphological changes in parasomnias, published between 01/01/2015 and 30/06/2024 and available during the search period, were included. We only looked at studies from the last 10 years to check the progress since early determinative studies [9, 21, 22, 26] and make sure we included the most up-to-date evidence. This is especially important in the quickly developing field of neuroscience, also helping to maintain a manageable scope for data extraction and synthesis, minimizing information overload, and enabling an in-depth analysis of high-quality literature [37, 38].

### Data extraction

MSB and VMC had independently searched for research papers from databases, and the accessed research reports were exported to EndNote X7 for further processing. The necessary data were extracted using a standardized data extraction format, which included the following items: first author, publication year, sample size, instrument used, results on localization, and morphological changes. All authors did a cross-check following the searches. There was further discussion to achieve consensus, and double extraction was made. Agreements and controversial reports of included studies were discussed.

Outcome parameters.

Brain-topographic and morphological changes shared by NREM parasomnias and specific ones in their subtypes. In this review, brain topography refers to any report on EEG and other imaging methods (MRI, functional MRI, SPECT, and PET); changes in local spectral powers, and brain networking connections. In addition, for this review, we defined morphological changes as any changes in brain parts’ morphology and volume.

### Quality assessment

The qualities of the articles were independently assessed by MSB and VMC using the Newcastle Ottawa scale. We used the Newcastle Ottawa scale for case-control and cohort studies. Case-control studies have been assessed for selection, comparability, and exposure. On the other hand, the included cohort studies had been assessed for selection, comparability, and outcome. The rest of the authors helped to come up with a consensus regarding disagreements between the two reviewers.

### Summary of the protocol

The registered protocol outlines a systematic review aimed at synthesizing evidence on the localization and morphological correlates of NREM parasomnias. The objectives include identifying brain localization and morphological changes in people with NREM parasomnias, and evaluating findings from neuroimaging (MRI, fMRI, PET) and EEG-based source localization studies. The protocol followed PRISMA guidelines and includes quality assessment using the Newcastle-Ottawa Scale for observational studies. We confirm that the review adhered closely to the pre-specified protocol. No deviations or amendments were made during the review process. 

## Results

### Study selection

**Fig. 1:**
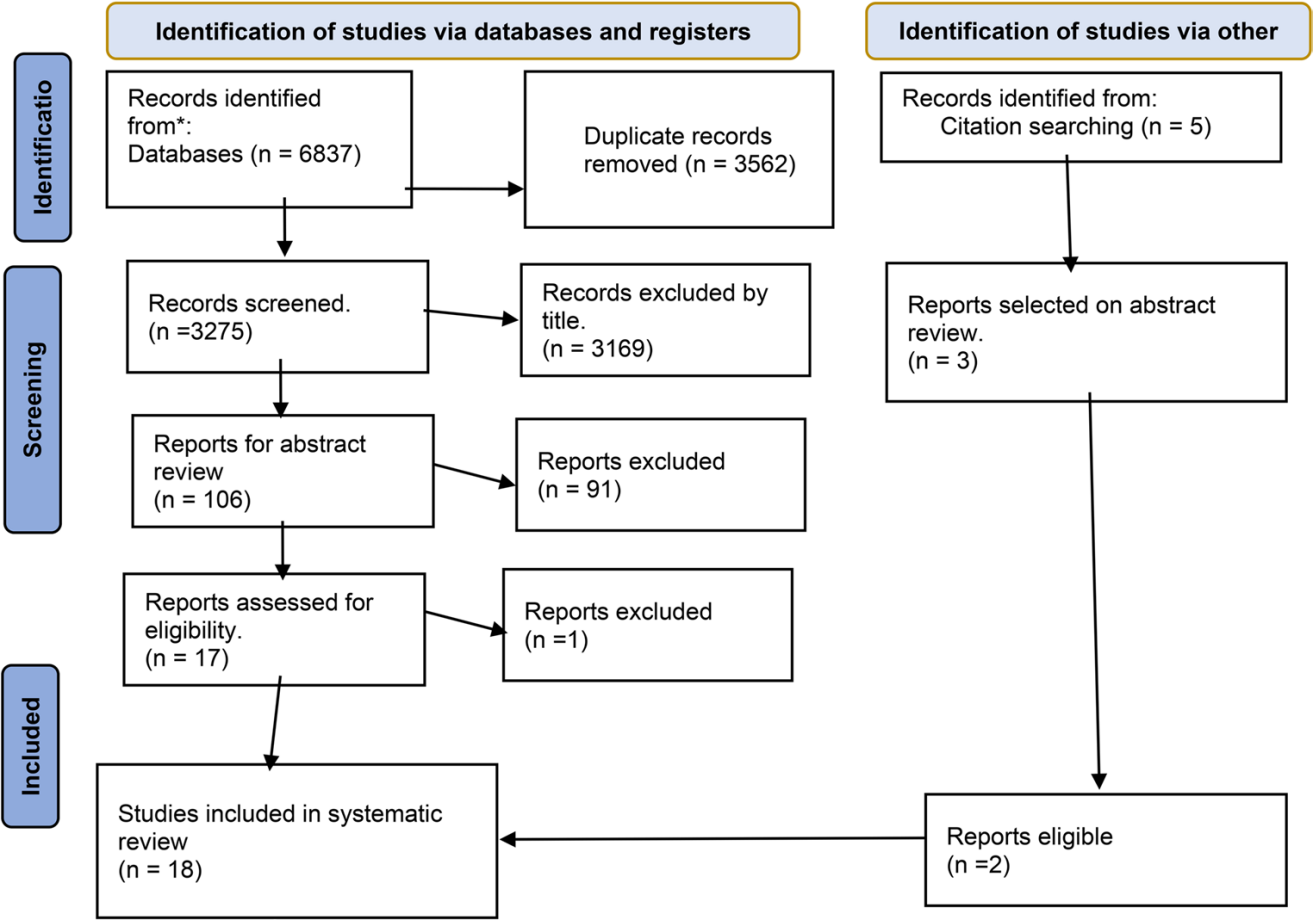
PRISMA flow diagram for the article selection process

A total of 6837 articles were identified from databases and side searches from references. After the removal of duplicates as well as only titles and abstracts, we found 107 full-text articles. Finally, 18 articles met our eligibility criteria for review. We excluded 3169, 91, and one record after title, abstract, and full text reviews, respectively. The excluded reports did not meet our predefined inclusion criteria, which were based on study design, relevance to our research question, and methodological quality. Specifically, some reports were excluded because they focused on conditions outside the scope of NREM parasomnias, lacked localization or morphological data (Fig. 1).

A total of 487 patients with NREM parasomnias and healthy controls have been involved in all studies. Most studies (83%) applied EEG/polysomnography, 2 SPECT, one MRI, and one exact low-resolution electromagnetic tomography (eLORETA) for their imaging. Half of the included studies were conducted in Italy and Canada (4 in Italy and 4 in Canada) (Table 2).

**Table 2:** Characteristics of included studies

Author/year	Study population	Sample size	Imaging technique	Country	Category
Carpentier, N. et al., 2020	Adult sleepwalking	59	EEG	Canada	Localization
Castelnovo A. et al., 2016	Adults with arousal disorder	30	256 channel EEG system	Italy	Localization
Dang-Vu, T. T. et al., 2015	Adult sleepwalking	22	99mTc-ECD SPECT	Canada	Localization
Desjardins M. et al., 2017	Adult sleepwalking	27	EEG	Canada	Localization
Desjardins M. et al., 2018	Sleepwalking	20	SPECT 99mTc-ECD scans	Canada	Localization
Dubessy, A. L. et al., 2017	Sexsomnia	17	VPSG	France	Localization
Ertaş, Fİ et al., 2021	Sleepwalking	28	Neuropack Sigma MEB-5504k	Turkey	Localization
Flamand M. et al., 2018	Confusional arousal	5	EEG	France	Localization
Heidbreder A. et al. 2017	Adult NREM parasomnias	28	3 Tesla MRI	Austria	Morphological changes
Januszko, P.et al., 2016	Sleepwalking	15	EEG neuroimaging (eLORETA)	Poland	Localization
Mangiaruga, A. et al. 2022	Sleep talking	26	EEG	Italy	Localization
Merli, E. et al., 2019	Sleep-Related Rhythmic Movement disorder and Sleep Terror	1	EEG	Italy	Localization
Miletínová, E. et al. 2023	Sleepwalking and sleep terror	16	hdEEG and fMRI	Czech Republic	Localization
Pani, S. M. et al., 2021	Sleep-related hypermotor epilepsy and NREM parasomnias	31	VPSG EEG	Italy	Localization
Rossi, J. et al. 2023	Sexsomnia, sleepwalking and sleep terror	105	VPSG EEG	France	Localization
Sarilar, A. C. et al. 2021	Sleepwalking, confusional arousal and sleep terror	39	PSG with additional EEG montages	Turkey	Localization
Cataldi J. et al. 2022	sleepwalking, sleep terror, confusional arousal	20	hdEEG	Switzerland	Localization
Cataldi J. et al. 2024	sleepwalking, sleep terror, confusional arousal	22	hdEEG	Switzerland	Localization

### EEG changes before and during DOA episodes

The findings are not consistent, sometimes contradictory, and may point to different regions. Desjardins et al. from Canada reported detailed results on the timing of EEG changes related to sleepwalking episodes. They found a significant increase in the spectral power of delta and theta frequency bands in the 20 s immediately preceding the episodes’ onset when compared to the 20-second segment occurring 2 min prior to each episode [39]. Congruently, Flamand et al. indicated an increase in delta activity, predominantly in the frontal regions, in the last few seconds before the onset of confusional arousal episodes [40].

An EEG spectral analysis of Merli et al. during the partial arousal of sleep terror episodes found higher slow-wave activity compared to the wakefulness preceding sleep (Fz-Cz), discriminating those partially aroused parasomnia-episodes from full wakefulness [41]. A similar intrusion of sleep (excess slow waves) into wakefulness was found by the nice high-density EEG (hdEEG) study of Cataldi et al. In people with DOA, they reported high amplitude, steep K-complex-like (type I) slow waves in frontal and central regions (reminiscent of hypersynchronous delta) preceding both NREM parasomnia episodes and non-parasomnia-related arousals from NREM sleep, followed by a concomitant increase in EEG beta activity. According to that study, parasomnia episodes evolved from “less activated” sleep periods, i.e., “deeper” sleep with more slow waves and less beta. In addition, the excess of slow waves was constituted by lower amplitude (type II) slow waves, suggesting a different arousal mechanism in ‘normal’ and ‘parasomnia-related’ arousals. Thus, an abnormal timing (and placement) of arousal-related slow wave synchronization processes could underlie the occurrence of NREM parasomnias [42]. A more recent study of the same group reported high-amplitude EEG slow waves in anterior cortical regions (1–4 Hz delta) and the activation of posterior cortical regions (26–34 Hz beta), preceding the episodes of DOA; a similar EEG pattern to the correlates of dreaming [43].

Januszko et al., using EEG current density imaging and eLORETA, found that sleepwalking episodes were preceded by sudden partial arousals (t >4.52; *p* < 0.05) with an increase of 24–30 Hz beta-3 in the cingulate motor area (Brodmann 33 and 24) [31] within the sleeping brain.

Castelnovo et al. performed hdEEGs with source localization in N2 and N3 sleep of sleepwalking and sleep terror patients, far from clinical episodes. They reported a significant decrease of slow wave power in central areas, notably the cingulate, motor/premotor/supplementary motor, the associative somatosensory and visual cortices as well as in the superior parietal lobule and the precuneus; (largely overlapping areas with those found to be activated during sleepwalking episodes); while there was higher slow wave power in the orbitofrontal, ventromedial prefrontal, the angular and the primary sensory cortices [15]. This study supported the existence of cortico-cortical sleep-state dissociation even far from clinical events in NREM and REM sleep as well as in wakefulness [15].

Also patients with sexsomnia were shown to present with cortico-cortical dissociation; concomitant slow (mostly frontal) and rapid (mostly temporal and occipital) EEG rhythms [44].

**Fig. 2:**
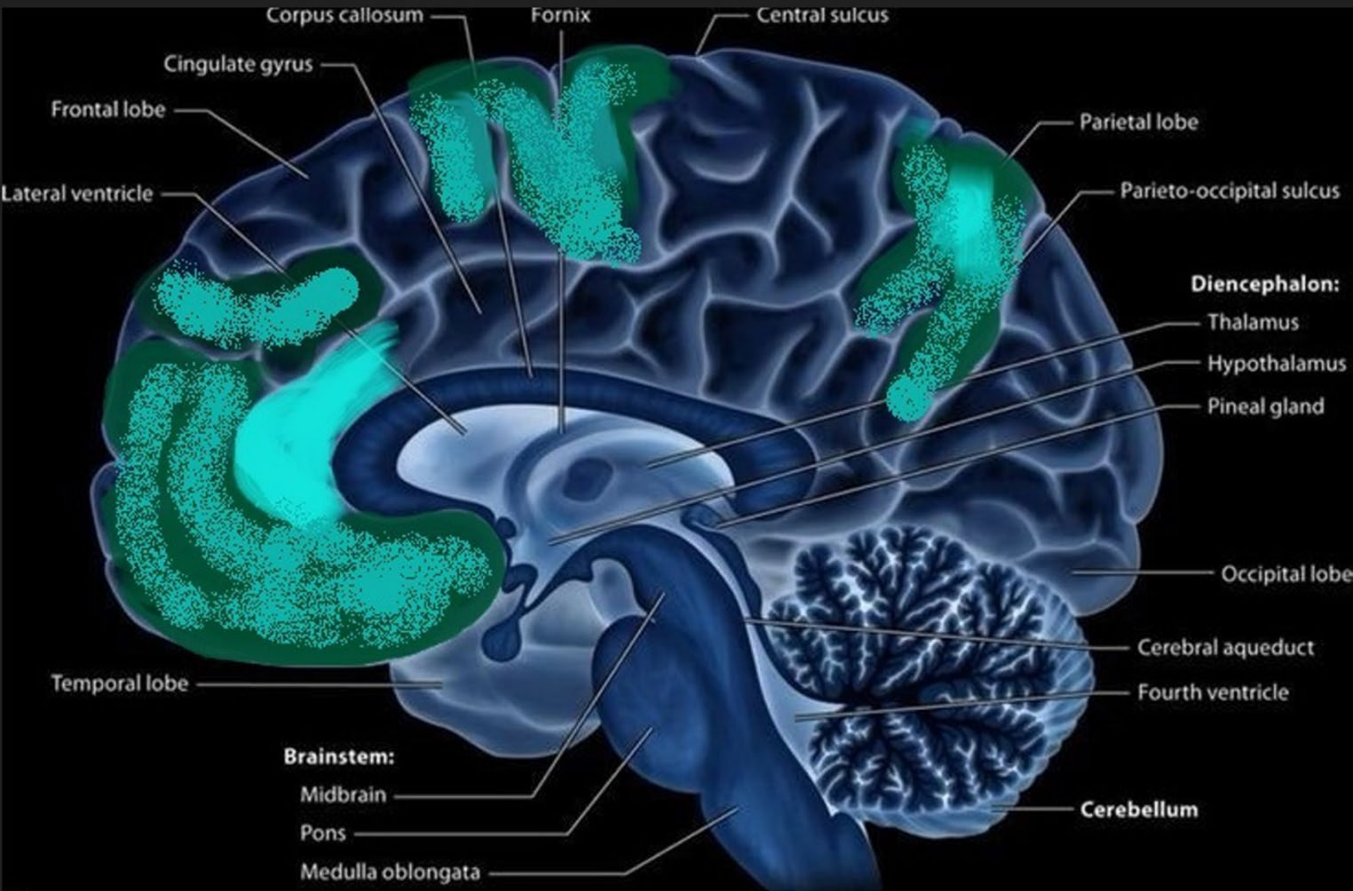
Schematic drawing of the medial surface of the brain. Brain regions shown by literature data to be activated within the sleeping brain before and during DOA episodes are highlighted in turquoise. The same regions are shown to produce slower waves (are more activated) than other cortical areas, in any vigilance state. Colour intensity reflects the consistency of literature data found about the highlighted region. It seems clear that the anterior cingulate gyrus is most involved

Flamand et al. found HSD involving a broad fronto-parietal network, especially the inferior frontal gyrus. They suggested that HSD might participate in the pathophysiological process, explaining the altered state of consciousness of patients during NREM parasomnia episodes [40] (Table 3) (Fig. 2).

**Table 3:** Summary of findings regarding localization and morphological changes in NREM parasomnias

	EEG changes immediately before and during episodes	Permanent morphology changes	Network changes	Changes of blood flow
DOA without specification	Decrease in slow wave power in a cluster of electrodes in centro-parietal areas, specifically during N2/N3 (*n* = 15) [15] An increase in delta and beta power over the postcentral gyrus and cuneus during awakening (*n* = 8) [45] Abnormally asymmetric sharply contoured K complexes in NREM-2 in seven out of 28 cases (*n* = 39) [46] Higher slope of the aperiodic component in DOA compared to SHE during N3 (*p* = 0.012) in the gamma frequency band (*n* = 16) [47] Large, steep, and “K-complex-like” slow waves in frontal and central brain regions, and a concomitant increase in high-frequency EEG (beta) activity [42] Higher slow wave activity and lower beta activity in frontal and central brain regions after episode-onset [42]. High-amplitude slow waves in anterior cortical regions and activation of posterior cortical regions [43]. Higher activation in the right medial temporal region before movement onset [43].	Significant decrease in grey matter- volume in the left dorsal posterior cingulate (BA23) and posterior midcingulate cortices (BA24) (*n* = 14) [48]	Enhanced connection between the motor and cingulate cortices during arousals unrelated to parasomnia-episodes in the beta frequency band and less connection within various cingulate segments and more connection between the thalamus and certain cortical regions, such as the occipital cortex (*n* = 8 DOA patients [45]	
Specific types of DOA				
sleepwalking	Increase of delta and theta powers in the 20 s preceding the episodes (*n* = 27) [39] Greater current density within the 24–30 Hz beta range (sudden partial arousal) in the cingulate motor area (Brodmann 33 and 24) before the onset of episodes (*n* = 15) [49] Sudden partial arousal from NREM sleep in the - cingulate motor area (*n* = 15) [49] More awakenings from N2 sleep and significantly lower SWA density in N2/N3 stages (*n* = 31) [50]		A lower delta band within 20 s preceding the episodes with lower functional connectivity for networks involving parietal and occipital regions (*n* = 27) [39] Higher alpha-band connectivity between frontal and parietal regions, and higher beta-band connectivity for symmetrical inter-hemispheric networks involving frontotemporal, parietal, and occipital areas (*n* = 27) [39]	Decreased regional cerebral blood flow bilaterally in the inferior temporal gyrus (*n* = 10) [51] Reduced regional cerebral perfusion in bilateral frontal regions, including the superior-, middle- and medial frontal gyri. Enhanced regional cerebral perfusion in the right parahippocampal gyrus (*n* = 10) [52]
sleep terror	Increased slow wave activity during the episodes, with respect to the wakefulness preceding sleep (*n* = 1)[45] Both before and during the episode, there is a clear predominance of the delta and theta bands (*n* = 1) [53]			
confusional arousal	An increase in delta activity in frontal regions in the last few seconds preceding the episodes (*n* = 19) [40]		Hypersynchronous delta activity immediately preceding episodes in medial and lateral frontoparietal cortices and the inferior frontal gyrus (*n* = 19) [40]	
sexsomnia	Evidence of cortico-cortical dissociation, including concomitant slow (mostly frontal) and rapid (mostly temporal and occipital) EEG rhythms (*n* = 17) [54] A higher N3 fragmentation index (*n* = 82) in sexsomnia and other DOA as well [54, 55]			
Sleep-talking	Decrement in theta and alpha power for verbal sleep talking, lateralized to the left hemisphere and localized in central-parieto-occipital channels (*n* = 13) [56]

### Connectivity changes

A study reported that sleepwalking and sleep terror patients showed enhanced connectivity in the beta-band between the motor and cingulate cortices and less connectivity within various cingulate segments during arousals unrelated to parasomnia episodes; in contrast, higher connectivity between the thalamus and certain cortical regions, such as the occipital cortex [45]. It has also been shown that a lower delta band preceding within 20 s the sleepwalking episodes, had associated with lower parieto-occipital alpha band-connectivity (*p* < 0.05), higher fronto-parietal (*p* < 0.05) and symmetric interhemispheric beta band connectivity (*p* < 0.05) in the frontotemporal, parietal and occipital areas (27). In another study, before the episodes, there was 1–1.5 Hz HSD activity involving a broad network in medial and lateral fronto-parietal cortices, as well as in the inferior frontal gyrus. In parallel, higher frequency activities increased in sensorimotor, orbitofrontal, and temporo-lateral cortices [40].

### Cerebral blood flow

Dang-Vu et al. found decreased cerebral blood flow in the bilateral inferior temporal gyri of sleepwalking patients compared to controls during wakefulness following a night of total sleep deprivation [50]. Desjardin et al. reported reduced regional cerebral perfusion during slow-wave sleep and resting-state wakefulness in sleepwalking persons compared to controls in the bilateral superior, middle, and medial frontal gyri. Reduced perfusion was found during slow wave sleep also in sleepwalkers’ left postcentral gyrus, insula, and superior temporal gyrus, compared with controls. During resting-state wakefulness, reduced cerebral perfusion also occurred in left parietal and temporal regions of sleepwalkers versus increased perfusion in the right parahippocampal gyrus [51].

Changes in brain morphology.

We found only one article published during the search period reporting permanent morphological changes in patients with mixed (sleepwalking, sleep terror, and confusional arousal) NREM parasomnias. There was a significant decrease in the volume of the left dorsal posterior cingulate (BA23) and posterior mid-cingulate cortices (BA24) in patients compared to controls, raising the possibility that those regions might be involved in pathophysiology of the condition [48].

## Discussion

We aimed to review shared brain localizations and morphological changes across NREM parasomnias as well as specific ones in their subtypes. The EEG changes described in the literature, were sorted to ones found in the waking periods of NREM parasomnia patients; changes in timely relation with the episodes, and those far from the episodes in sleep. The results found were highly inconsistent due to multiple types of patient selection (mixed or specific DOA populations) and methodological issues such as EEG sampling times (before, during, or between behavioural episodes); connectivity, spectral power, and eLORETA studies as well as heterogeneous imaging methods. Most publications have dealt with sleepwalking, some with mixed types of DOA, and a few with just specific types such as sleep talking, sexsomnia, confusional arousals, or sleep-related eating disorders.

During different DOA-episodes, the predominantly involved activated regions included the anterior cingulate and the motor cortex, confirming the early SPECT study of Bassetti et al. showing the highest increases of regional cerebral blood flow during sleepwalking in the anterior cerebellum and in the posterior cingulate cortex contrasting large frontal and parietal association cortices with less blood flow deactivated; in sleep. Additional regions of interest were the centro-parietal, visual, associative parietal, fronto-central regions, and the cuneus [29].

In certain studies, the EEG beta-connectivity increased between the motor and cingulate cortices and between frontal and parietal alpha frequency bands, as well as the thalamus and the occipital cortex, even during non-behavioural arousals [45], indicating permanently increased motor readiness, likely due to dysregulated cortical-subcortical interactions [54]. Conversely, reduced connectivity within cingulate segments suggested Default Mode Network (DMN) disorganization, potentially disrupting motor and cognitive regulation during NREM sleep [55].

The early stereo-EEG study of Terzaghi et al., evidencing the sleep-wake dissociation concept; local arousal of the motor and cingulate cortices versus increased delta activity in the frontoparietal associative cortices immediately before and during confusional arousal [28]; has been confirmed by the eLORETA study of Januszko et al. They have shown locally increased 24–30 Hz activity in the anterior cingulate cortex before several sleepwalking episodes of multiple patients [31], confirming “fluid boundaries” between sleep and wakefulness; local cingulate arousal within sleeping brains. The study of Castelnovo et al. has pointed out that roughly the same set of regions was affected by state-dissociation in NREM and REM sleep as well as in wakefulness, far from clinical episodes. The sleep-wake dissociation during waking has been confirmed by another study as well: DOA persons’ waking states were scattered by slow waves; suggesting permanently unstable state-boundaries in this group of disorders [45].

Therefore, we feel it justified to consider overall NREM parasomnias as ‘state-dissociation conditions’ characterized by a trait-like sleep-wake-dissociation; i.e., one occurring in each vigilance-state of the affected patient’. This could account for their unexpected neuropsychological changes and daytime sleepiness [45].

The results seem to converge to a central role of the cingulate gyrus. The anterior cingulate cortex is the main site of K-complex generation [25]. This region is linked to emotional processing, voluntary motor control, and decision-making and is closely interlinked with the salience network [56], which in turn may activate the hypothalamo-pituitary-adrenal axis, driving stress responses [57]. Therefore, its local (dissociated) activation in line with prevailing sleep in most parts of the brain may result in complex motor activities with partial or lacking awareness to the episodes; as well as emotional and fearful outbursts emerging in sleep terrors [58].

Certain studies have shown HSD just before or at the very onset of sleepwalking episodes [39, 40], possibly indicating a build-up of neural activity disrupting sleep architecture and triggering DOA episodes with sensory and spatial processing [28, 59]. The existence of HSD might confirm the hypothesis of DOA resulting from an imbalance of sleep-promoting and arousal processes [60, 61].

The reported increase in slow wave activity during the partial arousals of sleep terror episodes underscores the unique neural bases that distinguish parasomnias from ‘‘normal’’ arousals and typical sleep-wake transitions [41, 62].

A study on EEG differences in verbal versus non-verbal sleep talking found reduced theta and alpha power in the left centro-parieto-occipital regions, disrupting normal oscillatory rhythms and enabling speech-related processes during sleep [63].

Studies on cerebral blood flow point to the exposure of key areas in the generation of slow wave sleep and DOA episodes, as well as of those regions involved in impaired awareness and the reduced pain perception during sleepwalking episodes [21, 51, 64]. Those studies highlighting the hypo-perfusion in frontal and temporal areas and hyperperfusion in the parahippocampal gyrus confirm that sleepwalking involves complex, state-dependent brain dysfunctions as well as state-dissociations [65].

We found but a single morphological study in NREM parasomnia patients. In convergence with other imaging methods, it has revealed reduced grey matter volume in the left dorsal posterior cingulate and posterior mid-cingulate cortex [48]. Such cortical volume-reduction could be involved in the dysregulation of arousal and sleep-stage transitions, contributing to the wake-like motor activity with impaired consciousness in sleep [66, 67]. Additionally, structural abnormalities in these regions might hinder the normal suppression of the DMN during NREM sleep, promoting parasomnias [68].

While this single study identifies intriguing morphological alterations in individuals with NREM parasomnias, it requires confirmation by other, preferably systematic trials on higher number of DOA-patients. This highlights a significant gap in the literature and underscores the importance of future research using larger samples and multimodal imaging approaches to explore the neuroanatomical underpinnings of NREM parasomnia subtypes.

Limitations.

A major limitation of this review is the small number (just one) of studies investigating structural brain changes in individuals with NREM parasomnias. As such, any interpretations regarding brain morphology remain speculative and should be approached with caution.

Another notable limitation is the significant heterogeneity across the included studies, encompassing differences in study design, sample size, neuroimaging modalities (e.g., EEG, fMRI, PET), participant populations (heterogeneous patient population), as well as the timing of data acquisition relative to parasomnia episodes. This variability precluded a meta-analytic approach and limits the generalizability of our conclusions.

As a possible limitation of the study, language restrictions and database selection may have resulted in the omission of relevant studies published in non-English languages or those indexed in less commonly used databases. Another limitation might be that the study was conducted following the steps of the systematic review, through which we didn’t perform a quality appraisal for each step.

## Conclusions

NREM parasomnia episodes are evidenced to be underlined by a localised arousal of anterior frontal and cingulate regions in a sleeping brain. This is congruent with the finding that patients show slow wave deficits in roughly the same fronto-cingular regions, even far from clinical episodes in NREM and REM sleep and in wakefulness. This overall state-dissociation across vigilance states (a trait-like sleep-wake dissociation) seems to characterize NREM parasomnias. It might point to a weakness of sleep/wake-regulatory forces in this thalamo-cortical segment, or a local dysfunction of fronto-cingulate regions in parasomnia patients. The latter hypothesis could be supported by the morphology change of this region revealed by a single study, as well as by the slow-wave features (deeper sleep constituted by lower amplitude slow waves than in episode-free arousals of parasomnia patients) of the clinical episodes. Different NREM parasomnia subtypes may have shared dissociation features involving additional cortical regions.

The heterogeneity in reported imaging techniques and study populations as well as the timing of data acquisitions; have hampered our drawing general inferences. Addressing these factors and involving bigger patient populations in future research will be crucial to achieve a unified understanding of the underlying mechanisms and localisations.

Our review provided a novel synthesis of recent neurophysiological and neuroimaging findings that deepen our understanding of the localization and morphological correlates of NREM parasomnias and their subtypes. By integrating evidence from high-density EEG, source localization studies, structural and functional imaging, we highlight distinct patterns of cortical and subcortical involvement, particularly in cingulate, frontoparietal, limbic, and sensorimotor networks during parasomnia episodes. Our review also targeted specific subtypes such as sleepwalking and confusional arousals, suggesting still insufficiently subtype-specific neural circuits and vulnerabilities. Furthermore, emerging data on grey matter alterations and disrupted connectivity in key arousal regulation areas suggest that NREM parasomnias may reflect not only state instability but also trait-like morphological predispositions.

## Supplementary Information

Below is the link to the electronic supplementary material.

Supplementary Material 1

Supplementary Material 2

## Data Availability

The data that support the findings of this study are available from the corresponding author upon reasonable request at biresaw.mengesha@phd.semmelweis.hu.

## Abbreviations

CAP: Cyclic Alternating Pattern
DOA: Disorders of Arousal
DMN: Default Mode Network
EEG: Electroencephalography
eLORETA: exact Low-Resolution Electromagnetic Tomography
fMRI: Functional Magnetic Resonance Imaging
HSD: Hypersynchronous Slow Waves Discharge
MRI: Magnetic Resonance Imaging
NREM: Non-Rapid Eye Movement
PET: Positron Emission Tomography
PRISMA-p: Preferred Reporting Items for Systematic Reviews and Meta-Analyses
RBD: REM Sleep Behaviour Disorder
REM: Rapid Eye Movement
SHE: Sleep-related Hypermotor Epilepsy
SPECT: Single-Photon Emission Computed Tomography
SRED: Sleep-related eating disorder
99mTc-ECD SPECT: 99mTc-Ethyl Cysteinate Dimer SPECT
VPSG: Video-Polysomnography
